# Impact of Non-*Saccharomyces* Wine Yeast Strains on Improving Healthy Characteristics and the Sensory Profile of Beer in Sequential Fermentation

**DOI:** 10.3390/foods11142029

**Published:** 2022-07-08

**Authors:** Vanesa Postigo, Paula Sanz, Margarita García, Teresa Arroyo

**Affiliations:** 1 Department of Agri-Food, Madrid Institute for Rural, Food and Agriculture Research and Development (IMIDRA), El Encín, A-2, km 38.2, 28805 Alcala de Henares, Spain; paula.sanz9991@gmail.com (P.S.); margarita_garcia_garcia@madrid.org (M.G.); teresa.arroyo@madrid.org (T.A.); 2 Brewery La Cibeles, Petróleo 34, 28918 Leganes, Spain

**Keywords:** non-*Saccharomyces*, sequential fermentation, beer aroma, wine yeast

## Abstract

The use of non-*Saccharomyces* yeasts in brewing is a useful tool for developing new products to meet the growing consumer demand for innovative products. Non-*Saccharomyces* yeasts can be used both in single and in mixed fermentations with *Saccharomyces cerevisiae*, as they are able to improve the sensory profile of beers, and they can be used to obtain functional beers (with a low ethanol content and melatonin production). The aim of this study was to evaluate this capacity in eight non-*Saccharomyces* strains isolated from Madrid agriculture. For this purpose, single fermentations were carried out with non-*Saccharomyces* strains and sequential fermentations with non-*Saccharomyces* and the commercial strain SafAle S-04. The *Wickerhamomyces anomalus* strain CLI 1028 was selected in pure culture for brewing beer with a low ethanol content (1.25% (*v*/*v*)) for its fruity and phenolic flavours and the absence of wort flavours. The best-evaluated strains in sequential fermentation were CLI 3 (*Hanseniaspora vineae*) and CLI 457 (*Metschnikowia pulcherrima*), due to their fruity notes as well as their superior bitterness, body, and balance. Volatile compounds and melatonin production were analysed by GC and HPLC, respectively. The beers were sensory-analysed by a trained panel. The results of the study show the potential of non-*Saccharomyces* strains in the production of low-alcohol beers, and as a flavour enhancement in sequential fermentation.

## 1. Introduction

The brewing industry generally uses *Saccharomyces* yeasts for brewing, while non-*Saccharomyces* yeasts have been more characteristic of spontaneously fermenting beers such as Sour and Lambic beer styles [1].

During fermentation, the sugars present in the wort are totally or partially transformed into secondary metabolites such as aromas (higher alcohols, esters, sulphur compounds, acids, vicinal diketones), CO_2_, ethanol, glycerol, and melatonin by the yeasts. Some compounds also affect the antioxidant capacity of the beer, thus determining the final quality of the beer [2,3,4,5].

Likewise, over the last few years, the craft beer industry has been growing and with it the interest on the part of consumers for new beers with different properties [6]. This demand has led to pure culture and sequential fermentations with non-conventional yeasts to obtain beers with a low ethanol content, as well as functional and good organoleptic properties [7]. In this context, studies on wine are more abundant than on beer. Sequential or mixed fermentations in beer has been studied with species such as *Pichia kluyveri*, *Brettanomyces* spp., *Torulaspora delbrueckii*, *Hanseniaspora guilliermondii*, *Hanseniaspora opuntiae*, and *Lachancea thermotolerans* [8,9,10,11,12], and a production of esters and higher alcohols has been reported that would enhance flavours in beers [13]. However, due to the competition that can occur between *Saccharomyces* and non-*Saccharomyces* yeasts during fermentation, this has not yet been extensively studied [11,14,15].

Due to the low ethanol production of non-*Saccharomyces* yeasts, fermentative yeasts of the genus *Saccharomyces* are commonly used as starter cultures in both mixed and sequential fermentations. However, even with low ethanol production, around 1% (*v*/*v*), non-*Saccharomyces* yeasts are able to attenuate the wort flavour and provide characteristic aromas to the resultant low ethanol beer [9,12,16,17]. *Saccharomycodes ludwigii*, *Zygosaccharomyces rouxii*, and *Torulaspora delbrueckii* species have been evaluated mainly for the production of low ethanol beer, with an organoleptic profile widely accepted by consumers [9,12,18]. In addition, some non-*Saccharomyces* yeast strains can produce melatonin and antioxidant compounds that contribute to the functional properties to the final beverage [5,19,20]. Eight non-*Saccharomyces* yeast strains of the species *Hanseniaspora vineae*, *Hanseniaspora valbyensis*, *Hanseniaspora guilliermondii*, *Metschnikowia pulcherrima*, *Zygosaccharomyces bailii*, *Torulaspora delbrueckii*, and *Wickerhamomyces anomalus* isolated from Madrid agriculture were evaluated in pure and sequential culture conditions to assess their fermentative and sensory suitability. For beers fermented in pure culture, residual sugars, ethanol production, and sensorial properties were studied. In the case of beers brewed in sequential culture, parameters such as glycerol, bitterness, colour, SO_2_, lactic acid, VDKs, volatile compounds, melatonin, and antioxidant activity were also determined in addition to residual sugars and ethanol production.

## 2. Materials and Methods

### 2.1. Yeast Strains

Eight non-*Saccharomyces* strains belonging to seven different species were used in this study (Table 1). They were selected from previous studies [21] and are preserved under cryogenization at −80 °C in the Autochthonous Yeast Collection of the Madrid Institute for Rural, Food and Agriculture Research and Development (IMIDRA, Madrid, Spain). They were isolated from different resources (from grapes, must, wine, vineyards, and cellars) belonging to D.O. “Vinos de Madrid”.

Fermentation trials were performed using the *Saccharomyces cerevisiae* commercial strain SafAle S-04 (Fermentis, Lesaffre, Marcq-en-Barœul, France) as the control.

In addition, to determine the purity of the yeast strains used, they were previously analysed by amplification of the 5.8S rRNA gene and the two ribosomal internal transcribed spacers, using the primer pair ITS1/ITS4 [22]. The resulting PCR product was analysed by restriction enzymes (Hae III, Cfo I and Hinf I) [23].

### 2.2. Propagation and Fermentation

Precultures were grown in 100 mL Erlenmeyer flasks with 30 mL of YPD broth (2% yeast extract, 1% bacteriological peptone, and 2% glucose; all *w*/*v*) (Condalab, Madrid, Spain), and orbital agitation (120 rpm) at 28 °C for 24 h. One-litre fermenters were used to carry out the fermentation trials, containing 900 mL of sterilised wort and with an inoculum concentration of 10^6^ cells mL^−1^ (for both non-*Saccharomyces* and *Saccharomyces* yeast strains). For sequential fermentations, non-*Saccharomyces* yeast strains were inoculated and fermented for five days and then *Saccharomyces* S-04 was added. In addition, monocultures for both strains, non-*Saccharomyces* and S-04, were prepared as control sets. The wort used for the study was elaborated in La Cibeles brewery with the following characteristics: pH, 5.73; gravity, 11.92° Plato and 1.047 g cm^−3^; free amino nitrogen, 239.67 ppm; bitterness, 37.43 IBU.

All fermentations were performed at triple determination, with rotatory shaking at 18 °C. Fermentation was performed by automatic control of lost weight each hour until no change in weight could be measured for two consecutive days. Fermented beers (green beers) were mixed with 7 g L^−1^ of glucose for bottled conditioning, and stored at 20 °C for one month. After this period, beers were stored at 4 °C for maturation. The viability of the investigated yeasts was measured before bottling by the direct microscopic method with a Thoma counting chamber and the methylene blue procedure. The resultant beers were sensorially and analytically studied.

### 2.3. Monitoring of Beer Fermentation: WL Medium and Quantitative Real-Time PCR

WL medium and real-time PCR were used for monitoring yeast populations during brewing fermentations. Samples were taken daily, and the number of the total cultivable yeasts was determined by plating the samples in selective Wallerstein Laboratory Nutrient (WLN) agar (Condalab, Madrid, Spain), while the total populations were analysed by quantitative real-time PCR.

Colonies grown on WLN are usually cream to dark green, depending on the yeast species [24]. Yeasts were enumerated by spread-plating 100 µL of the sample (serial diluted). WLN agar plates were incubated for five days, at 28 °C.

#### Primer Design and qPCR

The primers used for qPCR are shown in Table 2. For this study, two primers were designed for the strains CLI 194 and CLI 1028, based on the sequencing of the D1/D2 conserved domain of the large subunit (26S) rDNA gene. The Primer3Plus program was used for this purpose (https://primer3plus.com/, accessed on 18 May 2022) as well as the OligoAnalyzer™ Tool, to verify its suitability (https://eu.idtdna.com/pages/tools/oligoanalyzer, accessed on 18 May 2022). All primers used in the study were synthesized by Metabion (Planegg, Germany).

DNA was extracted from 1 mL of daily beer sample, centrifuged (14,000 rpm for 3 min), and the supernatant was discarded. The pellet was then washed with sterile distilled water and centrifuged again. Extraction was carried out using the DNeasyPlant Mini Kit (QIAGEN, Valencia, CA, USA), whereby the pellet was first resuspended in 700 μL of AP1 buffer, and then transferred to a 2 mL microcentrifuge tube containing 1× *g* of 0.5 mm diameter glass beads. The tubes were shaken in a mixer mill (Retsch GmbH, Haan, Germany) for 3 min at maximum speed and then centrifuged at 10,000 rpm for 1 min. Finally, the supernatant was transferred to a sterile tube as described in the kit manufacturer instructions for DNA purification.

The qPCR was performed on a QuantStudio 5 Dx Real-Time PCR System (Thermo Fisher Scientific Inc., Waltham, MA, USA). Reactions were performed in optical-grade 96-well plates (Applied Biosystems, Life Technologies, Johannesburg, South Africa). DNA was amplified in a reaction containing 12.5 μL FastStart Universal SYBR Green Master Mix (Roche Diagnostics GmbH, Mannheim, Germany), 5 μL of purified DNA, 0.75 μM of each forward and reverse primer, and 6 μL PCR water (25 μL in total reaction). Each reaction was performed in triplicate with the following real-time PCR parameters: (i) 95 °C/10 min; (ii) 40 cycles of 95 °C/30 s, 60 °C/1 min; and 72 °C/30 s. The signals produced (threshold cycle, Ct) by the serial dilution in YPD for each strain were analysed to build standard curves.

### 2.4. Analysis of the Beers

Beers obtained in sequential fermentation were analysed after maturation for lactic acid concentration (in a range of 150–3500 ppm), bitterness (in a range of 5–100 IBU—International Bitterness Unit), colour (in a range of 1–100 EBC—European Brewing Convention), SO_2_ (in a range of 1–30 ppm), and vicinal diketone production: diacetyl and 2,3-pentanodione (VDKs) (in a range of 0.05–2 ppm). For this purpose, the CDR FoodLab (BeerLab software) (https://www.cdrfoodlab.com/, accessed on 18 May 2022) was used, verified by the international reference analysis laboratory CampdenBRI (https://www.campdenbri.co.uk/, accessed on 18 May 2022).

Before each analysis, the samples were degassed through a filter of grade 2 V (Whatman, Maidstone, UK).

### 2.5. Determination of Sugar Concentration, Glycerol, and Ethanol Content

The concentrations of ethanol, glycerol, maltotriose, maltose, glucose, and fructose were determined before bottling using an HPLC equipment Dionex Ultimate 3000 (Thermo Scientific, Waltham, MA, USA), equipped with a quaternary pump, an autosampler, a column compartment provided with a temperature controller, and a 520 refractive index detector (ERC). An ionic exclusion column, Rezex ROA-Organic Acid H+ (8%), 150 × 7.8 mm (Phenomenex) was used. The temperature separation was at 60 °C and the mobile phase was H_2_SO_4_ 0.005 M, with a constant flow rate of 0.6 mL min^−1^ [29]. Samples were previously filtered through a 0.22 μm filter and the injection volume was 10 μL. Concentrations were estimated based on appropriate calibration curves with an R^2^ value > 0.9881.

### 2.6. Volatile Compound Analysis

Volatiles (33 major aromatic compounds of the following groups: higher alcohols, esters, acids, acetaldehydes-ketones, lactones, and phenols) were determined according to the method from Ortega et al. [30] based on liquid-phase microextraction with dichloromethane (DCM) (Panreac-Applichem, Barcelona, Spain). After the microextraction, the volatile compounds were identified by gas chromatography. A gas chromatograph 6850 (GC-FID, Agilent Technologies, Inc., Santa Clara, CA, USA) equipped with a flame ionization detector was used. Volatiles were separated in a DB-WAX capillary column (60 m × 0.32 mm i.d. and 0.5 μm film). The flow rate of carrier gas helium was set at 2 mL min^−1^. The oven temperature was held at 40 °C for 5 min, and then increased at a rate of 3 °C min^−1^ to a final temperature of 200 °C. Injector and detector temperatures were 200 °C, with splitless injection.

For the determination of the main aromatic compounds, four internal standards related to the different compounds (2-butanol, 4-methyl-2-pentanol, 4-hydroxy-4-methyl-2-pentanone, 2-octanol) were used (200 ppm each). The concentration of the different compounds was determined by calibration curves for each of the compounds (R^2^ = 0.9861–0.9969).

### 2.7. Melatonin Production and Antioxidant Capacity of Beers

Melatonin extraction from the matured beers was performed by solid-phase extraction (SPE) with standard RP-18 PP tubes (Agilent Technologies, Inc., Santa Clara, CA, USA). The process consisted of preparing and conditioning the columns with 2 mL of methanol (Scharlab, Barcelona, Spain) and 5 mL of bidistilled water. Subsequently, 500 μL of beer was added, and the impurities were washed with 2 mL of bidistilled water and eluted with 2 mL of methanol [31]. The samples were then dried under a stream of nitrogen and in a thermoblock at 80 °C. Finally, they were reconstituted with 300 μL of methanol and 700 μL of mobile phase (formic acid (0.1%)/Acetonitrile (95:5)) (HPLC grade; Carlo Erba, Italy/Panreac-Applichem, Barcelona, Spain). The reconstituted samples were also passed through filters (0.22 μm) before HPLC analysis.

The chromatographic separation was performed using a Waters 600 HPLC controller system which consisted of an autosampler (Waters 717 plus) and a multifluorescence detector (Waters 2475). The fluorescence detector recorded wavelengths of 270 nm for excitation and 372 nm for emission. The separation was performed using a ZORBAX Eclipse Plus C18 column (Agilent Technologies, Inc., Santa Clara, CA, USA) at a 30 °C column temperature. The mobile phase comprised a mixture of 0.1% formic acid in water and acetonitrile (95:5), and the isocratic flow was 1 mL min^−1^. The volume of injection was 10 μL. The concentration of melatonin was determined by using a linear calibration curve (R^2^ > 0.9856) [32,33,34].

The antioxidant capacity of the beers was analysed with the e-BQC lab device (Bio-quochem, Asturias, Spain, www.bioquochem.com, accessed on 27 June 2022). This device measures the redox potential and expresses it in micro-Coulombs (μC) [35]. For the analysis of antioxidant activity, the TEAC (Trolox Equivalent Antioxidant Capacity) assay was used with a solution of 6-hydroxy-2,5,7,8-tetramethylchromane-2-carboxylic acid (Trolox 8 mM L^−1^ in 5% methanol and pH 4.5). The Trolox calibration curve (Q1, R^2^ = 0.9974; Q2, R^2^ = 0.9876; QT = Q1 + Q2) was developed using the measurement of e-BQC versus Trolox concentration (μmol L^−1^). Therefore, the antioxidant activity of the beers was expressed as millimoles of Trolox equivalents per litre (mmol TE L^−1^).

### 2.8. Sensory Analysis

Sensory evaluation was performed on bottled beers samples (matured as described in Section 2.2) using a paired comparison test [36] with a trained and experienced panel (five male, five female). The paired comparison test consisted of the evaluation of beers brewed in pure culture (*Saccharomyces* and non-*Saccharomyces*) against beer fermented in sequential culture, in order to finally determine the preference for one or the other. Descriptive sensory analysis was performed using a panel of 10 assessors, who were trained to detect specific flavours in beer (diacetyl, acetaldehyde, DMS, bitter, lactic acid, butyric acid, isovaleric acid, earthy, H_2_S, clove, geraniol, grainy, papery, light-struck, indole) (Siebel Institute of Technology, Chicago, IL, USA) according to the EBC method 13 [37,38,39]. The chosen attributes to describe the products were divided into three groups: appearance (colour, foam retention), aroma (fruity, phenolic, banana, hop, yeast, worty) and taste (alcohol, sweet, bitterness, salty, acidic, astringency, effervescence, slickness, warmth, body). Assessors evaluated these attributes from absence to presence using a scale from 0 to 5 points. The tasting was conducted in a dedicated tasting room (white room, with no distracting elements and equipped with individual tasting chambers). The main average values obtained for the different parameters evaluated in the beers were plotted on a radar graph.

### 2.9. Statistical Analysis

The means and standard deviations (SD) of the sequential beers were determined based on triplicate fermentations and are represented as mean ± SD. Experimental data were subjected to one-way analyses of variance (ANOVA) and Tukey post-hoc tests at a significance level of *p* < 0.05. To determine the significant correlations in the data, Pearson’s tests were performed. Principal component analysis (PCA) of the by-products (volatile compounds, melatonin, antioxidant capacity, lactic acid, colour, bitterness, VDKs, and SO_2_) was performed to visualise the beer samples in an n-dimensional space identifying the direction in which the variables contributed to the discrimination of the samples between groups. The statistical analysis was carried out using the software RStudio 4.1 (Integrated Development for R. RStudio, PBC, Boston, MA, USA).

## 3. Results

### 3.1. Main Characteristics of Beers

The sequential fermentations were initially performed with the single non-*Saccharomyces* strain and six days later the *Saccharomyces cerevisiae* S-04 strain was inoculated. The inoculum concentration for the non-*Saccharomyces* strain and the S-04 strain was 10^6^ cells mL^−1^. Single fermentations (pure cultures) of non-*Saccharomyces* and S-04 were also included in the study for sensory analysis. Viable cells before bottling ranged between 1.2 and 9.7 × 10^6^ cells mL^−1^ for pure cultures, and between 3.5 × 10^4^ and 1.1 × 10^7^ cells mL^−1^ for sequential cultures, depending on the yeast strain.

The main parameters analysed in the matured beers are presented in Table 3. One of the most important characteristics when fermenting beer is that the selected yeast is able to ferment the sugars present in the wort (glucose, fructose, sucrose, maltose, and maltotriose) [40,41]. The study strains were able to ferment glucose and fructose, while only strains CLI 902, 7A-3A (*T. delbrueckii*), and CLI 1028 (*W. anomalus*) were able to ferment glucose, fructose, and sucrose, as seen in previous studies [21]. The ability to ferment sucrose would indicate that these strains have the genes coding for the production of the enzyme invertase, which is necessary for converting sucrose to glucose and fructose [42,43]. Furthermore, none of the yeast strains fermented maltose or maltotriose, maltose being the most abundant sugar in the wort, the fermentation of which can be repressed by glucose since its presence represses the transcription of enzyme-coding genes such as maltase [44,45,46]. As expected, and as seen in previous studies [21], none of the eight strains studied showed fermentation behaviour comparable to that of the commercial strain of *S. cerevisiae* in pure culture.

As expected, ethanol concentrations were higher in those fermentations where strain S-04 was involved, ranging from 5.11 to 5.67% (*v*/*v*) for sequential fermentations and 5.93% (*v*/*v*) for S-04 pure culture. However, for single-culture fermentations of non-*Saccharomyces* strains, ethanol production never exceeded 1.25% (*v*/*v*), confirming their lower fermentative power. The ability to ferment sucrose was also reflected in ethanol production for the strains CLI 3, CLI 194, CLI 457, CLI 512, and CLI 691, whose values ranged from 0.61 to 0.73% (*v*/*v*). The sequential cultures with *T. delbrueckii* strains and *S. cerevisiae* produced beers with different ethanol levels. These were significantly lower for strain 7A-3A (2.15% (*v*/*v*)), associated with higher residual maltose levels. The fact that the S-04 yeast was not able to complete fermentation could be due to competition for nutrients in the medium or between the cells of the two species [47]. The study carried out by Canonico et al. with mixed fermentation of *S. cerevisiae* and *T. delbrueckii* showed that the residual maltose was higher in the fermentation, where the percentage of *T. delbrueckii* cells was twenty times higher than that of *S. cerevisiae* [9]. This behaviour has also been observed in wine, where competition between cells decreases the concentration of viable cells, thus reducing the ethanol content [48].

Regarding glycerol production, this compound has an effect on the mouthfeel and body of the beer, thus influencing its flavour [49,50,51]. Generally, its production is related to cell growth, ethanol production, as well as sugar consumption, which is why the values found in pure culture fermentations were between 1.08 and 1.34 g L^−1^, while in sequential cultures, the values increased from 2.81 to 4.28 mg L^−1^, being higher than those obtained by the control strain S-04 (2.89 mg L^−1^). *Metschnikowia*, *Hanseniaspora*, and *Torulaspora* wine yeasts are characterised by high glycerol production [52].

The data for the analytical compositions of the beers produced were analysed with CDR Foodlab and are reported in Table 4. One of the main characteristics defining the appearance of beer is its colour. The malts are not the only ones responsible for this, but it is also dependent on the amount of melanoidins produced during the malting process. The wort recipe used in this study was always the same, thus the variations observed in colour are dependent on the study strain. Some studies have shown that top-fermenting yeasts produce darkening and oxidation of melanoidins, leading to beers with higher absorbance values [53]. The values obtained range from 10.33 to 15 EBC, with 10 EBC for the control strain S-04.

During the brewing process and more particularly during the boiling process, the α-acids of hops are transformed into iso-α-acids, which will contribute to the bitterness of the beer [54,55]. The hops used in the brewing process remained constant throughout the study, thus the differences observed are strain-dependent. Several studies suggest that the metabolism of the yeast cell wall causes α-acid molecules to adhere to them, depositing them at the bottom of the fermenter, thus reducing the bitterness of the beer [56,57,58,59,60]. The initial bitterness value of the wort was 37.43 IBU, which generally decreased during fermentation, with values ranging from 22.85 to 35.20 IBU, and 16.35 IBU for the S-04 strain. Therefore, sequential fermentation contributed to obtaining more bitter beers compared to the commercial strain S-04. Similar behaviour was also observed between strains, since for *T. delbrueckii* (CLI 902 and 7A-3A), the values were 26.3 and 26.10 IBU, respectively, and for *W. anomalus* (CLI 1028), 35.2 and 35.5 IBU, respectively. Bitterness showed a positive correlation of r = 0.63 and *p* < 0.05 with antioxidant activity.

For the commercial strain, lactic acid values of 304.25 ppm were obtained. Sequential fermentation generally contributed to an increase in lactic acid concentration (322 to 395.5 ppm), but in no case were threshold levels (400 ppm) exceeded [61]. However, strain CLI 691 (*Z. bailii*) showed a reduction in production (234 to 233 ppm), as did strain 7A-3A (*T. delbrueckii*) (264 ppm), although fermentation was not finished. Lactic acid is formed by yeast metabolism from pyruvate, the main substrate being glucose. However, the physiological role of lactic acid production and the molecular mechanisms remain unknown [62].

Sulphur dioxide (SO_2_) can act as a bleaching agent, oxygen scavenger, antimicrobial agent, reducing agent, and enzyme inhibitor, and for this reason it has been commonly used as an additive in the food industry [63]. Yeasts not only produce H_2_S in terms of sulphur compounds but can also produce small amounts of SO_2_. The maximum levels of SO_2_ accepted in beer usually depend on how it is regulated in different countries, with the maximum being 10 ppm in Spain. There were no major differences between the values obtained by the different strains (between 1 and 1.45 ppm), which are within the legally established levels. Furthermore, these values did not exceed the threshold (20 ppm) [64] above which they would produce unpleasant aromas. Due to its antioxidant activity, some authors [63,65] suggest that low SO_2_ concentrations may contribute to the stabilisation of beer over time, as it can react with compounds that give rise to stale taste (acetaldehyde and trans-2-nonenal) [2].

Vicinal ketones (VDKs) are considered to be diacetyl and 2,3-pentanedione, and their presence is undesirable for beer quality, as they produce unpleasant aromas and flavours in lager and ale beers. During fermentation, yeast cells excrete an intermediate of valine biosynthesis, α-acetolactate, which is decarboxylated to diacetyl. Both compounds impart a strong caramel and butterscotch aroma to beer, whose flavour thresholds are very low, 0.15 ppm and 0.9 ppm respectively [66]. In this study, the values of VKDs are below this threshold (0.10 to 0.39 ppm), thus no beer would impart these undesirable aromas. VDKs were correlated with total aldehyde/ketone aromatic compounds (r = 0.73 and *p* < 0.05).

### 3.2. Monitoring Sequentially Inoculated Beer Fermentations

Sequential fermentations of non-*Saccharomyces* and *S. cerevisiae* yeasts showed a generally similar fermentation progress, except for the fermentation with the 7A-3A strain (*T. delbrueckii*), which manifested intermediate fermentation capacity. The final fermentation capacity of the beers fermented sequentially with CLI 194 (*H. vineae*) and CLI 902 (*T. delbrueckii*) was also noteworthy, as it exceeded that of S-04 (57.3 g CO_2_ L^−1^), with values of 67.75 and 76.65 g CO_2_ L^−1^, respectively. However, the rest of the strains, after inoculation with the *S. cerevisiae* strain, showed a CO_2_ production similar to that of the S-04 strain in pure culture. The same results were observed by Holt et al. in sequential fermentations with non-*Saccharomyces* [10].

**Figure 1:**
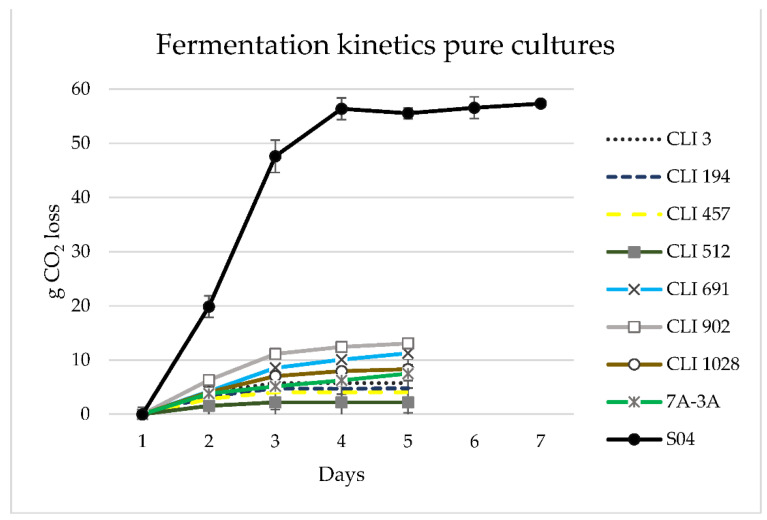
Fermentation kinetics in 1 L single fermentations for the eight studied strains. Each value is the mean of three trials ± standard deviations expressed in g CO_2_ lost per day.

The fermentations of pure cultures showed the slowest kinetics compared to the S-04 strains, as they produced a lower amount of CO_2_ (Figure 1), thus confirming the incomplete attenuation of sugars in the wort.

**Figure 2:**
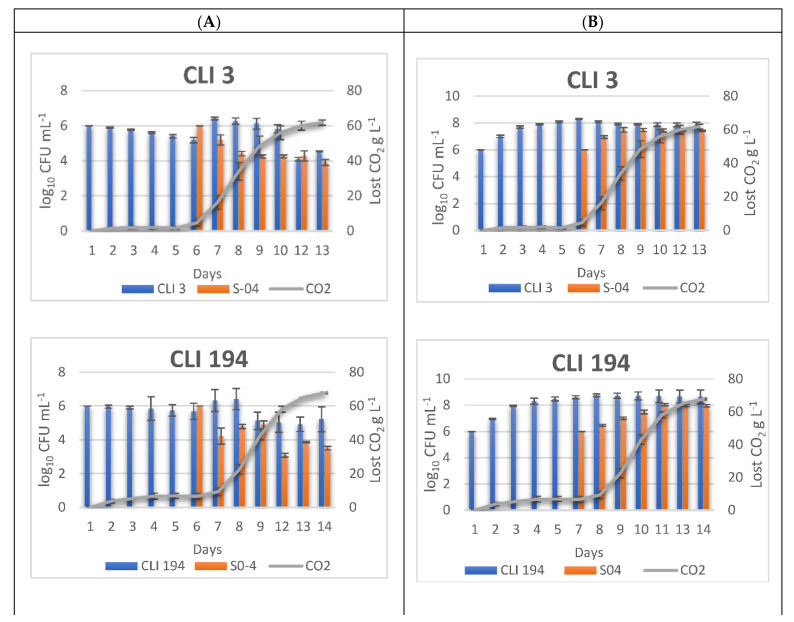

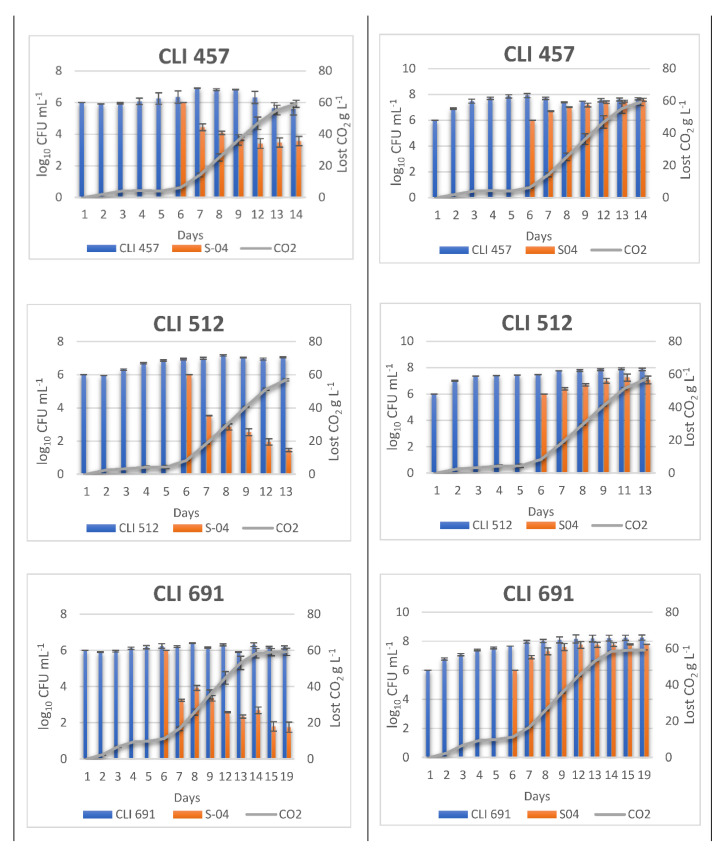

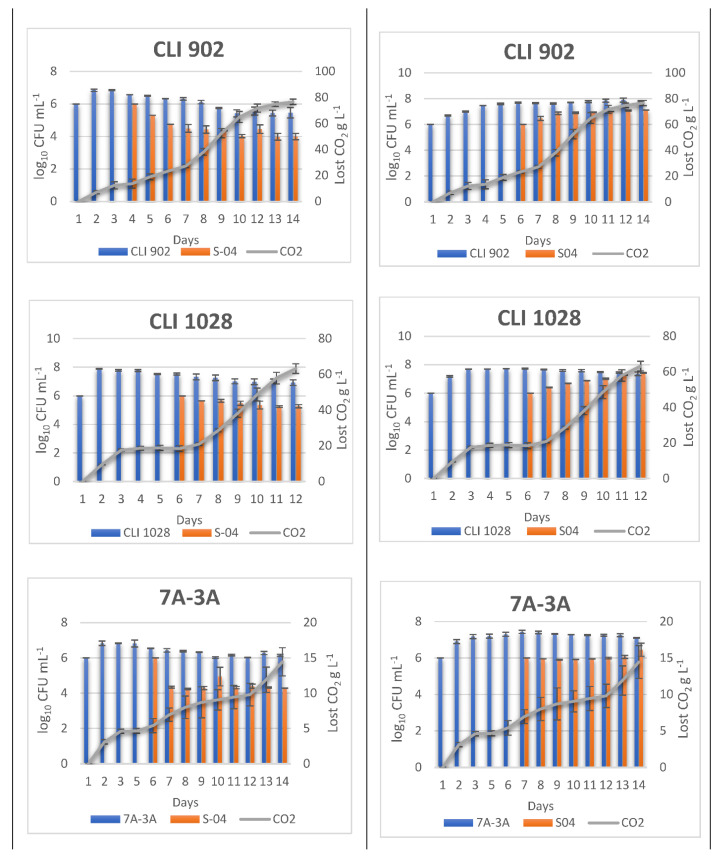

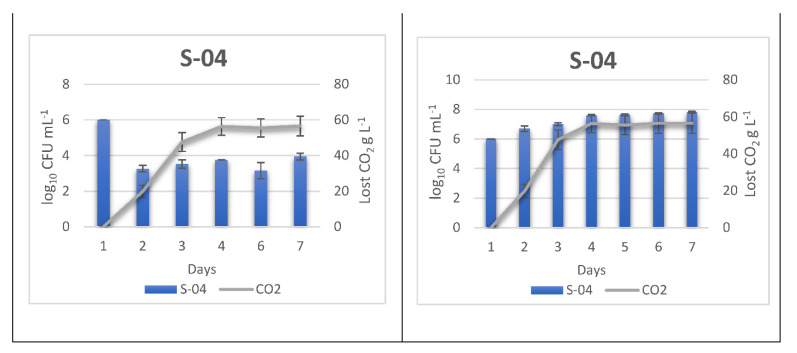
Fermentative kinetics and yeast monitoring of the different beers fermented sequentially with S-04 strain as control in WL medium. (**A**)—Yeast viability in sequential fermentation analysed with WL medium. (**B**)—Total yeast population in sequential fermentation analysed by qPCR. The data shown are the average ± standard deviations of three independent samples.

The populations of the sequential fermentations were monitored on the one hand by using a simple differential agar medium (WL nutrient medium) for the total culturable cells (Figure 2A) and on the other hand by qPCR for the total populations (Figure 2B).

In terms of cell viability in sequential cultures (Figure 2A), for *Hanseniaspora* strains CLI 3 and CLI 194, a decrease in cell population was observed prior to the addition of *Saccharomyces* yeast, which, once inoculated, again produced a further increase in non-*Saccharomyces* cells. Likewise, with beer initially inoculated with strain CLI 457 (*M. pulcherrima*), an increase in the non-*Saccharomyces* yeast population was also observed after inoculation with S-04. However, this was not observed with strains CLI 512 (*H. guilliermondii*) and CLI 691 (*Z. bailii*), where the populations at the time of inoculation of S-04 remained constant for the rest of the fermentation. The fact that the population level of non-*Saccharomyces* yeasts remains constant suggests that these strains may contribute to the final chemical and sensory attributes of the beer. Likewise, a large decrease in the *Saccharomyces* viable population was observed for these fermentations, with a final concentration of 28.75 and 57.5 CFU mL^−1^, respectively, compared to those obtained in the other *Hanseniaspora* fermentations (CLI 3 and CLI 194), which were between 3.25 × 10^3^ and 8.03 × 10^3^ CFU mL^−1^. In contrast, in a study carried out by Bourbon-Melo et al. on sequential fermentations with *Hanseniaspora*, once the *Saccharomyces* yeast was inoculated, the population of non-*Saccharomyces* yeast decreased [8]. Sequential fermentations carried out with strains CLI 902, 7A-3A (*T. delbrueckii*) and CLI 1028 (*W. anomalus*) showed a gradual decrease in the population of viable non-*Saccharomyces* cells. The same is true for strain S-04 in these cases, where the decrease could be due to the killer activity of the toxins produced by this species (*W. anomalus*) against some yeasts and moulds [67,68,69] or the production of some compounds such as ketones, organic acid esters, and acetonitrile that strongly induce aneuploidy in *S. cerevisiae* [70,71]. In addition, several studies with *S. cerevisiae* have shown that it can accumulate antimicrobial peptides on the cell surface, which can lead to the death of some non-*Saccharomyces* species during cell-to-cell contact [52,72].

The evolution of the total cell population of the sequential fermentations was determined by qPCR analysis of DNA extracted from 1 mL of the daily beer samples taken in triplicate. The standard curves (Table 5) were determined for each strain grown on YPD and serially 10-fold diluted from 10^8^ cells mL^−1^ to 10^2^ cells mL^−1^. Each primer used only exhibited specificity for the corresponding strain. In addition, strains CLI 902 and S-04 were studied in beer wort to determine the influence of the beer matrix on the efficiency of the qPCR system. It was observed that there were no significant differences (*p* < 0.05) between the qPCR efficiency in beer wort and in YPD medium (data not shown).

The growth kinetics of the different non-*Saccharomyces* strains achieved a range from 2.72 × 10^7^ to 8.11 × 10^7^ cells mL^−1^ for strains CLI 457, CLI 512, CLI 691, CLI 902, CLI 1028, and 7A-3A before the *Saccharomyces* S-04 was inoculated (sixth day), while the range was 1.99 × 10^8^ to 4.2 × 10^8^ cells mL^−1^ for the CLI 3 and CLI 194 strains. At the end of fermentation (day 13–14), the population levels remained stable, or in some cases increased (CLI 194, CLI 512, CLI 902, CLI 902) (from 1.3 × 10^7^ to 4.35 × 10^8^ cells mL^−1^). On the other hand, once the *Saccharomyces* S-04 yeast was inoculated, the total population remained constant or decreased slightly concerning the initial population of 10^6^ cells mL^−1^, depending on the strain. The behaviour of strain 7A-3A (*T. delbrueckii*) and S-04 in the sequential culture is noteworthy, since, once the *Saccharomyces* yeast was inoculated, the total yeast population remained constant until the end of fermentation, despite the fact that fermentation had not been completed. As mentioned above, this behaviour on the part of both strains (7A-3A and S-04) could be due to substrate competition and cell-to-cell contact, where *S. cerevisiae* could produce metabolites that inhibit non-*Saccharomyces* cells [48]. Furthermore, the fact that *T. delbrueckii* strain 7A-3A is not completely inhibited also suggests a high fermentative power, which implies a higher competitiveness of *T. delbrueckii* against *S. cerevisiae* strain S-04 in the wort [9,26].

### 3.3. Volatile Components in Beers

The use of non-*Saccharomyces* yeasts to improve the organoleptic characteristics of beers is becoming increasingly widespread, with sequential or mixed fermentation being the most commonly used techniques [9,10]. For this reason, the present study focused on the brewing of sequentially fermented beers in order to evaluate mainly the volatile compounds produced during fermentation.

The sequential fermentations showed aromatic profiles with significant differences, even between the same species, and when compared to the single fermentation with the control strain *S. cerevisiae* S-04 (Table 6). Differences were mainly observed in the higher alcohols, which increased for most of the strains studied, in the increase of some esters such as ethyl butyrate and diethyl succinate, and in the increase of some fatty acids (butyric, hexanoic, octanoic, and decanoic acid). The rest of the compounds remained at similar concentrations or slightly decreased for some strains. Within the aromatic compounds produced by yeasts, the most important groups are the higher alcohols as they are the most abundant compounds, and esters, as they have low threshold levels, which makes them important compounds for the definition of beer quality [3].

Higher alcohols impart freshness, floral aromas, as well as pleasant and desirable warming character compounds to beer when the total concentration of these compounds is below 300 mg L^−1^. The maximum concentration of higher alcohols was reached by the sequential fermentation carried out with the strain *T. delbrueckii* CLI 902 (229.76 mg L^−1^), being one third higher than those obtained by the control strain in pure culture (141.83 mg L^−1^). In addition, strains CLI 194, CLI 457, CLI 691, and CLI 1028 also exceeded these values. The concentration obtained by the *H. valvyensis* CLI 194 strain (189.91 mg L^−1^) is noteworthy since, despite having been described as a low producer of higher alcohols, its concentration is a quarter higher than that of S-04 [73,74]. On the other hand, the values obtained by the other two *Hanseniaspora* strains (CLI 3 and CLI 512) are lower than those of the control strain. This could be because one of the last steps of the Ehrlich pathway, where higher alcohols are produced from amino acids [3], may not be found in the genus *Hanseniaspora*, as it lacks the aryl-alcohol dehydrogenases necessary to reduce aldehydes to higher alcohols [75]. The two most important alcohols produced in beer are isoamyl alcohol and isobutanol (fusel alcohol), whose thresholds are 70 and 100 mg L^−1^ respectively [10,76]. These thresholds were only exceeded for isoamyl alcohol (alcoholic, vinous, sweet flavours) in fermentations with strains CLI 194, CLI 457, CLI 691, CLI 902, CLI 1028, and S-04. As for isobutanol, the highest concentrations were obtained in the sequential fermentation with *M. pulcherrima* (CLI 457), which has been previously reported as a high isobutanol producer [77]. Fermentations with the *W. anomalus* CLI 1028 yeast strain showed higher values than the pure culture of S-04. The strain *W. anomalus* has also been described as a good producer of β-phenylethanol, with a concentration obtained by strain CLI 1028 of 40.86 mg L^−1^, compared to 34.53 mg L^−1^ for the control strain. The same happened with the *T. delbrueckii* strains, where, in addition to differing in the fermentation process, the results obtained were also different. The strain *T. delbrueckii* exhibits the ability to produce β-phenylethanol (sweetish and floral flavours) and isoamyl alcohol [3], with concentrations of 67.23 mg L^−1^ and 113.04 mg L^−1^, respectively, found in this study.

Regarding ester production, in general, all strains contributed to increasing some esters concentration, with CLI 194 and CLI 691 as the highest producers. However, only the thresholds for isoamyl acetate (1.2 mg L^−1^, banana flavour) [78] for strains CLI 457 and CLI 691 (1.19 and 1.83 mg L^−1^, respectively) and diethyl succinate (1.2 mg L^−1^, berry flavour) [79] for strain CLI 194 (4.60 mg L^−1^) were exceeded. In fermentation carried out with strain CLI 457 (*M. pulcherrima*), despite exceeding the threshold, isoamyl acetate concentrations were reduced compared to the pure culture of S-04 (1.60 mg L^−1^). The *M. pulcherrima* yeast has been described as a good producer of isoamyl acetate [69,80,81] which is why the levels in the sequential fermentation were kept high. On the contrary, for strain CLI 691 (*Z. bailii*), these concentrations were higher, and thus this strain may have contributed to this increase even though it has not been previously described as a producer of high isoamyl acetate concentrations [18,82]. Diethyl succinate is formed during the esterification of succinic acid with alcohol, which is commonly found in aged beers and gives a berry flavour [83,84]. Diethyl succinate was not detected in the pure culture of the control strain S-04, however, it was detected in the sequential fermentation with strain CLI 194, so its formation can be attributed to this strain.

Although in general, the concentrations found for the different higher alcohols and esters did not exceed their thresholds levels, it should be mentioned that the presence of these volatile compounds can have a synergistic effect, thus providing a positive effect on the taste of the beer. Furthermore, the presence of esters at concentrations close to their thresholds may mean that small changes in their concentration can lead to a large variation in the taste of the beer [85,86].

Fatty acids can in many cases be responsible for undesirable aromas in beer when their thresholds are exceeded. Hexanoic, octanoic, and decanoic acids are responsible for the so-called caprylic flavour when their perception threshold is exceeded (8, 15, 10 mg L^−1^, respectively) [87], however, in none of the beers obtained in sequential fermentation were these thresholds exceeded. On the other hand, short-chain fatty acids such as butyric acid and isovaleric acid, which usually increase during fermentation [88], did exceed the thresholds in some of the beers analysed. Butyric acid is responsible for “cheesy” or “sickly” off-flavours in beer, with a threshold level of 2 mg L^−1^. All beers fermented sequentially produced concentrations higher than those of strain S-04, where in addition strains CLI 3, CLI 457, CLI 691, and 7A-3A exceeded the threshold levels. The old cheese, stale or sweaty aromas are due to the compound isovaleric acid, thus certain concentrations may make its presence in beer undesirable (2.5 mg L^−1^) [61]. This threshold was exceeded by strains CLI 1028, 7A-3A, and S-04, however, with the exception of strain CLI 1028 whose values were higher (6.90 mg L^−1^) than those of S-04 (4.16 mg L^−1^), for the rest of the fermentations it was significantly reduced. Although detection levels of these compounds were detected in several beers, these aromas were not subsequently found in tastings.

The main ketones analysed were diacetyl and acetoin. The species *T. delbrueckii* stands out as it is characterised by a high production of diacetyl which may not be reduced during bottle conditioning [7]. This fact could be observed in our study, as all beers were analysed after at least one month of maturation and the diacetyl threshold values were only exceeded in beers brewed with *T. delbrueckii* strains CLI 902 and 7A-3A.

The sequential fermentations also showed that all strains, except 7A-3A, produced similar concentrations of guaiacol. Phenolic off-flavours (POFs), such as guaiacol, give the beer a clove-like aroma. More specifically, guaiacol has a smoky flavour and a very low threshold (3.88 ppb) [86]. Despite being considered as undesirable aromas for certain beers, they are characteristic of other styles such as wheat beer, being one of its main aromas [89]. The highest concentrations obtained were by *W. anomalus* strain CLI 1028 (0.23 mg L^−1^), which is almost twice as high as for the control strain culture (0.12 mg L^−1^). Therefore, this strain would be a good candidate to produce beers with a phenolic profile.

### 3.4. Antioxidant Capacity

Antioxidant compounds can inhibit an oxidative reaction. This is because they are able to decrease molecular oxygen levels, and eliminate the free radicals that initiate and propagate the chain by chelating metals or decomposing peroxides [90,91]. Likewise, the antioxidant capacity of beer has positive effects on human health, as it can increase plasma antioxidant and anticoagulant activity and improve blood lipid levels, provided it is consumed in moderation [92,93].

The antioxidant capacity of beers fermented sequentially by the different strains is reported in Table 7.

The control strain S-04 produced total antioxidant capacity (Qt) levels of 11.18 mmol TE L^−1^, which were mostly exceeded by the sequentially fermented beers (11.56 to 13.70 mmol TE L^−1^), except for strains CLI 194 and 7A-3A (10.92 and 9.63 mmol TE L^−1^, respectively). Individually, the differences between the production of fast-acting antioxidants (Q1), which are considered more potent than slow-acting antioxidants (Q2), as well as being oxidised first, do not show large variations between beers, as do the slow-acting antioxidants. The values obtained were higher than those found in other studies, such as that of Granato et al. where the antioxidant capacity of beers ranged from 424.77 to 10,508.47 μmol TE L^−1^ [19]. These antioxidant capacity values can also vary according to the method used for their analysis, as values between 3.70 and 29.11 mmol TE L^−1^ can be obtained using the ORAC method. The oxygen radical absorbance capacity (ORAC) test is based on the absorbance capacity of oxygen radicals, thus measuring the decrease in fluorescence emission [94,95]. Another method is using ferric-reducing antioxidant power, the principle of which is the determination of the reduction of a ferric-tripyridyltriazine complex to its ferrous, coloured form in the presence of antioxidant components, with values of 3125 μmol of Fe^2+^ L^−1^ found in an ale beer. These values were also similar to those obtained in previous studies for *Saccharomyces* yeasts fermented under the same conditions of wort, temperature, and stirring [96].

### 3.5. Melatonin Production

**Figure 3:**
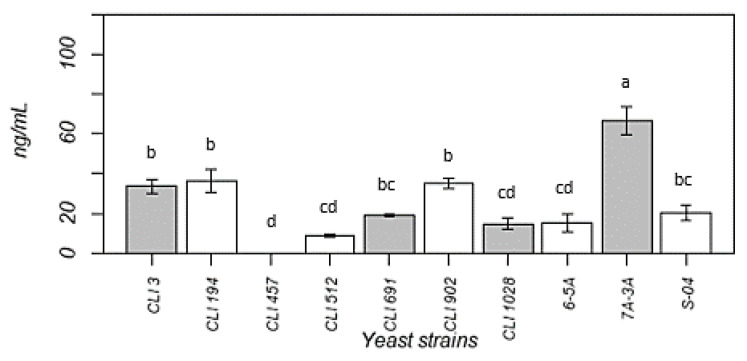
Melatonin production in beers fermented sequentially and in S-04 control strain, expressed in ng mL^−1^. The data shown are the average of three independent samples. Different letters next to the bars indicate a significant difference (Tukey tests: *p* < 0.05).

Melatonin is a hormone that regulates the sleep cycle as well as circadian and seasonal rhythms, acts as an immunostimulatory agent, and possesses antioxidant properties. Microorganisms have the ability to produce melatonin, including yeasts during the fermentation process. For this reason, a moderate consumption of beer can provide health benefits, and it is therefore considered a functional food [20]. Figure 3 represents the melatonin content obtained by the different strains fermented sequentially.

Melatonin levels found in the beers were higher than those of S-04 (20.41 mg mL^−1^) for four strains (CLI 3, CLI 194, CLI 902, and 7A-3A) (from 33.63 to 66.57 ng mL^−1^), with the melatonin found in the sequential fermentation carried out by the *T. delbrueckii* 7A-3A strain being particularly remarkable, since despite not having finished fermentation, its levels were three times higher than those of S-04 (66.57 ng mL^−1^). Some studies suggest that melatonin production by the different strains may be affected by the origin where the yeast was isolated or by its use, as adaptation mechanisms to different fermentation media may modify these melatonin production mechanisms. In this case, no melatonin production was detected in beer fermented with strain CLI 457 [97,98]. Melatonin production by strain S-04 in the fermentation with CLI 457 might have been inhibited by the increase in the non-*Saccharomyces* yeast population after the inoculation of *Saccharomyces* strain. Studies carried out by Maldonado et al. and García-Moreno et al. showed a positive relationship between ethanol levels and the concentration of melatonin found in beer [20,99]. However, in this study the correlation was negative (r = 0.76 and *p* < 0.05). Likewise, for these studies, melatonin levels in lager beers analysed by ELISA were lower. If we take into account the melatonin content in other types of food such as bread, tomato, or yoghurt, with values of 28.9, 138.1, and 126.7 pg mL^−1^ respectively, the concentration in the beers analysed would be above these values.

### 3.6. Sensory Analysis

**Figure 4:**
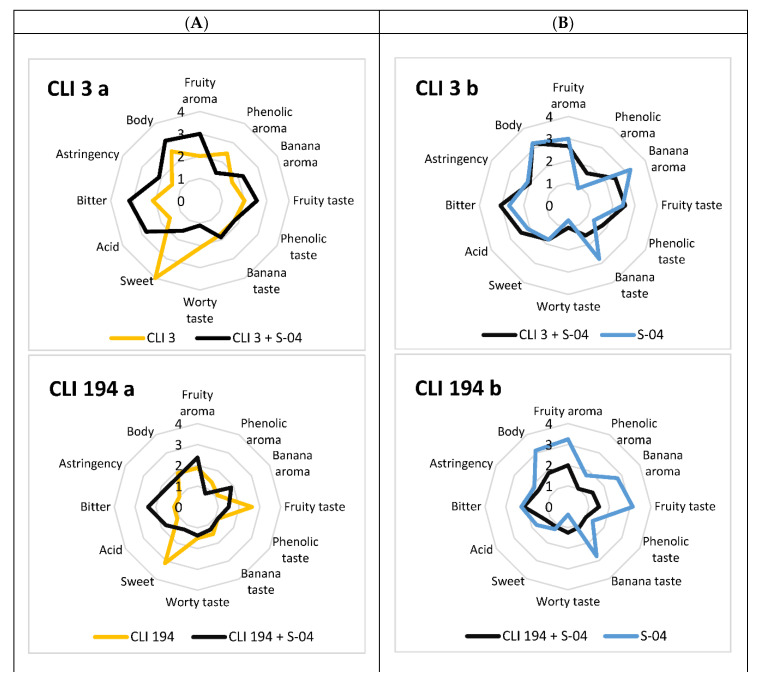

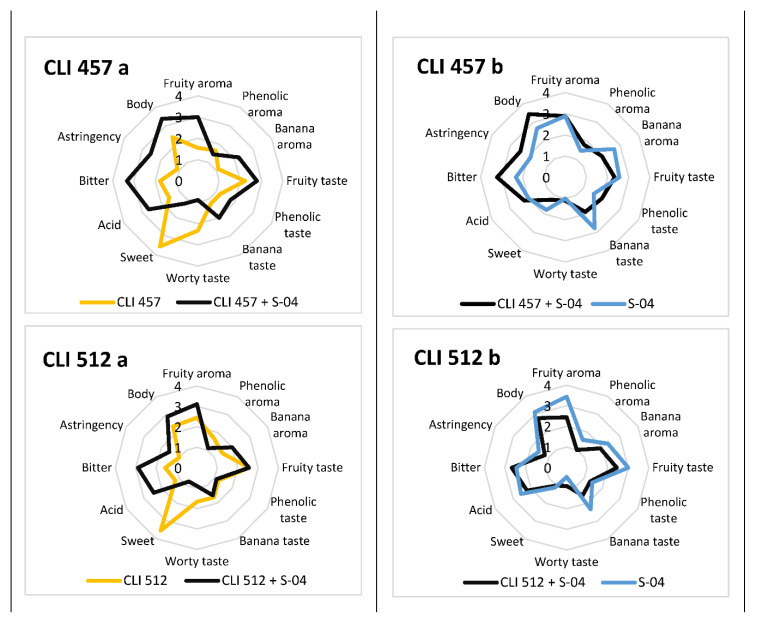

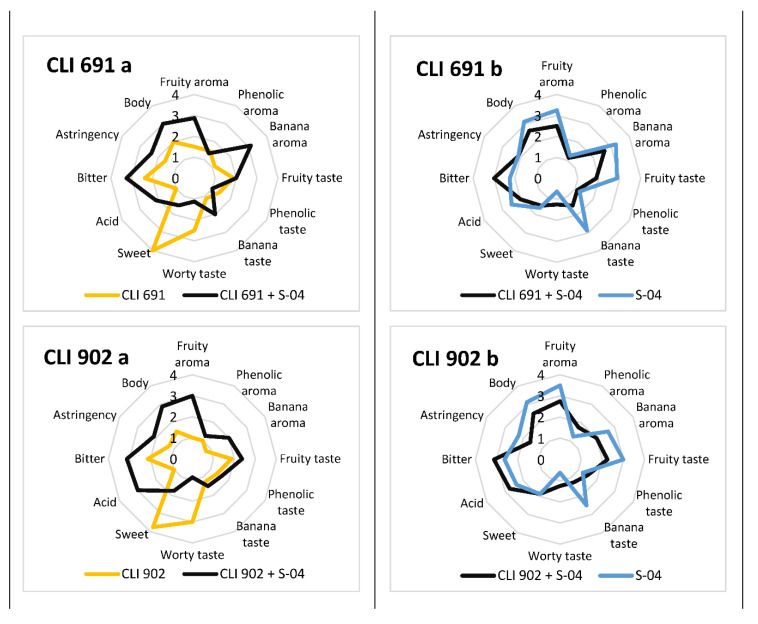

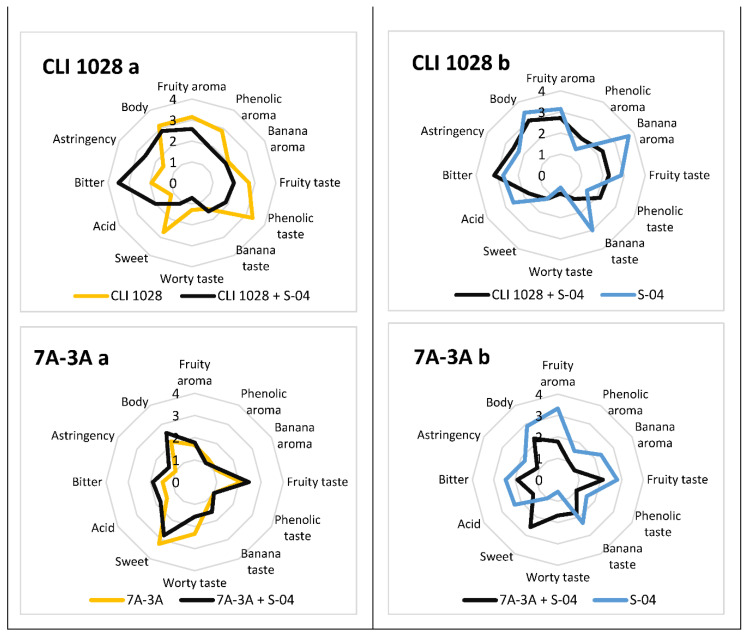
Sensory analyses of the different beers fermented in pure culture and sequential culture using a pared comparison test. (**A**) Sensory analyses of non-*Saccharomyces* pure cultures compared to sequential cultures. (**B**) Sensory analyses of sequential cultures compared to S-04 single culture.

The beers obtained in pure and sequential fermentations were sensory-analysed and are represented in a radar chart in Figure 4 using a paired comparison test.

From a general point of view, the sequential fermentations showed a good evaluation by the tasting panel. However, the fermentation carried out with the species *W. anomalus* (CLI 1028) is worth mentioning, as the pure culture was rated better than the sequential culture and the pure culture of S-04. Likewise, strains CLI 3 (*H. vineae*) and CLI 457 (*M. pulcherrima*) also stood out for their outstanding evaluation in sequential fermentation.

Non-*Saccharomyces* pure cultures were mainly characterised by sweetness and a worty flavour, due to incomplete fermentation of sugars. They also have common characteristics such as a phenolic character in some cases (CLI 1028) or quite prominent fruit characters, especially for strains CLI 194, CLI 512, and CLI 1028. Therefore, the *W. anomalus* strain was the best-evaluated in terms of pure cultures, since, despite not having finished fermentation, this strain did not have an accentuated sweetness and worty flavour, but did have a great fruity and phenolic contribution, with a slight bitterness and a medium body. The strain *W. anomalus* is a common yeast used for adding flavour to beer [100]. Therefore, it would be suitable for the production of beer with a low ethanol content.

Sequential fermentations were characterised by stronger fruity notes, banana flavours (mainly due to the S-04 strain), and higher bitterness and body. In this case, the best-evaluated fermentations were those carried out with strains CLI 3 (*H. vineae*) and CLI 457 (*M. pulcherrima*). As shown in other studies in beer and wine, *H. vineae* strains are characterised by enhancing the fruity characters of beer [11,101,102]. It also showed medium-high levels of body, bitterness, and acidity, making it preferable to the S-04 strain. On the other hand, the CLI 457 strain in sequential fermentation also showed a fruity character and more balanced phenolic and banana flavours than the control strain S-04, with slight acidity and no sweetness, which was observed in the analyses. As in other studies carried out with *M. pulcherrima* [103,104], for which the total ester concentration is not high, the sensory analysis shows it to be a yeast preferred by panellists and considered for use in sequential fermentation, as it improves the organoleptic characteristics of the beer. Therefore, with these results we can determine that some non-*Saccharomyces* strains can increase flavour diversity in sequential fermentation.

As has been seen in several studies, aromas undergo synergistic or antagonistic interactions, known as the “matrix effect”, thus changing their perception and the final taste of the beer even if the concentrations of these aromas are below the threshold [10,105,106,107,108,109]. Therefore, these interactions can make a beer desirable or undesirable, depending on the style, which makes sensory analysis one of the most important aspects when selecting a yeast strain.

### 3.7. Principal Components Analysis (PCA)

**Figure 5:**
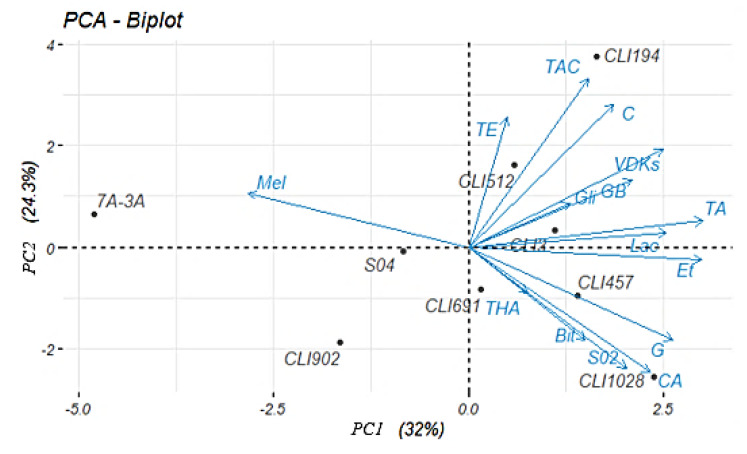
PCA-biplot of the beers fermented sequentially and the different parameters analysed. Each beer is the average of three samples. Mel, melatonin; Bit, bitterness; GB, γ-butyrolactone; Gli, glycerol; CA, antioxidant capacity; Et, ethanol; TA, total fatty acids; THA, total higher alcohols; TE, total esters; TAC, total aldehydes/ketones; Lac, lactic acid; G, guaiacol; C, colour.

Differences between the different variables (melatonin, bitterness, γ-butyrolactone, glycerol, antioxidant capacity, ethanol, total fatty acids, total higher alcohols, total esters, total aldehydes/ketones, lactic acid, guaiacol, colour, SO_2_, VDKs) separating the yeast strains were determined by multivariate principal component analysis (PCA) (Figure 5). Principal Components (PC 1 and PC 2) explain 56.3% of the system variance. All the variables analysed, except melatonin, were placed on the positive axis of PC 1. The *Hanseniaspora* yeast strains (CLI 3, CLI 194, and CLI 512) were grouped into positive values of PC 1 and 2, positively correlating with total esters, total aldehydes/ketones, total fatty acids, γ-butyrolactone, lactic acid, VDKs, and glycerol. On the other hand, strains CLI 457 (*M. pulcherrima*), CLI 691 (*Z. bailii*), and CLI 1028 (*W. anomalus*) were placed in the negative values of PC 1. In that quadrant the variables total higher alcohol, guaiacol, ethanol, SO_2_, bitterness, and antioxidant capacity were found. However, strains CLI 902 and 7A-3A (*T. delbrueckii*), despite being in the negative zone of PC 1, as well as commercial strain S-04, which was located near the melatonin variable, did not show a clear orientation towards any group of compounds.

## 4. Conclusions

Yeast selection is an important part of brewing an innovative beer, with non-*Saccharomyces* yeasts being the yeasts from which the greatest benefit can be obtained. The data obtained in the present study help to better understand the fermentative capacity, as well as the aromatic and sensory potential, of some native non-*Saccharomyces* yeast strains under pure and sequential culture conditions.

The fermentation carried out with the CLI 1028 strain of *Wickerhamomyces anomalus* showed a preference over the pure culture by the tasting panel, as it showed a better balance in the organoleptic characteristics of the beer, as well as pleasant flavours and a low perception of sweetness and wort flavour, characteristic of beers that have not finished fermentation. Furthermore, the fact that the fermentation was not completed meant that the final alcohol concentration was 1.25% (*v*/*v*). For this reason, this yeast could be used to produce low ethanol beers.

Many non-*Saccharomyces* yeast species are not able to ferment maltose, which is why they are used in sequential or mixed fermentations as they can contribute to unique flavour profiles. For this purpose, the best-evaluated yeasts were CLI 3 (*Hanseniaspora vineae*) and CLI 457 (*Metschnikowia pulcherrima*). These beers were characterised by a greater body and balance and fruity aromas and flavours, especially strain CLI 457, as the perception thresholds for isoamyl alcohol and isoamyl acetate were exceeded.

Likewise, the selected yeasts produced the highest levels of antioxidative capacity. The strains CLI 3 and CLI 1028 also produced melatonin during fermentation. All this suggests that these beers can be considered functional beers within moderate consumption.

In conclusion, beers brewed with non-*Saccharomyces* yeasts in sequential fermentations and pure cultures show important characteristics relevant to brewing.

## Figures and Tables

**Table 1:** Yeast strains used in single and sequential fermentation.

Yeast Strain	Species
CLI 3	*Hanseniaspora vineae*
CLI 194	*Hanseniaspora valbyensis*
CLI 457	*Metschnikowia pulcherrima*
CLI 512	*Hanseniaspora guilliermondii*
CLI 691	*Zygosaccharomyces bailii*
CLI 902	*Torulaspora delbrueckii*
CLI 1028	*Wickerhamomyces anomalus*
7A-3A	*Torulaspora delbrueckii*

**Table 2:** Primer sequences used for quantitative real-time PCR analysis.

Yeast Strains	Primer Name	Sequence 5′–3′	Reference
*Hanseniaspora vineae* (CLI 3)	CESP-F	ATCGAATTTTTGAACGCACATTG	[25]
	HUV-R	AACCCTGAGTATCGCCCACA	
*Hanseniaspora valbyensis* (CLI 194)	HV1-F	GCAGCTCAAAGTGGGTGGTA	This study
	HV1-R	GAGGCGAGTGCATGCAAAAA	
*Metschnikowia pulcherrima* (CLI 457)	MP2-F	AGACACTTAACTGGGCCAGC	[26]
	MP2-R	GGGGTGGTGTGGAAGTAAGG	
*Hanseniaspora gilliermondii* (CLI 512)	Hauf 2L	CCCTTTGCCTAAGGTACG	[27]
	Hauf 2R	CGCTGTTCTCGCTGTGATG	
*Zygosaccharomyces bailii* (CLI 691)	ZBF1	CATGGTGTTTTGCGCC	[28]
	ZBR1	CGTCCGCCACGAAGTGGTAGA	
*Torulaspora delbrueckii* (CLI 902, 7A-3A)	Tods L2	CAAAGTCATCCAAGCCAGC	[27]
	Tods R2	TTCTCAAACAATCATGTTTGGTAG	
*Wickerhamomyces anomalus* (CLI 1028)	WACLI2-F	ATTGGCGAGAGACCGATAGC	This study
	WACLI2-R	TTGCCATCCGAATCGATGCT	
*Saccharomyces cerevisiae* (S-04)	SC1	AAAACTCCACAGTGTGTTG	[27]
	SC2	GCTTAAGTGCGCGGTCTTG	

**Table 3:** Residual sugars, glycerol, and alcohol content in beer elaborated from pure and sequential fermentations analysed by HPLC.

Yeast Strains		Residual Fermentable Sugars (g L^−1^)				Glycerol (g L^−1^)	Ethanol % (*v*/*v*)
		Maltotriose	Maltose	Glucose	Fructose		
CLI 3	PC	13.68 ± 0.00 ^a^	63.80 ± 0.22 ^a^	0.25 ± 0.02	1.02 ± 0.00 ^a^	1.21 ± 0.01 ^f^	0.62 ± 0.17 ^e^
	SC	11.05 ± 2.33 ^abc^	1.16 ± 0.10 ^e^	0.23 ± 0.02	0.17 ± 0.00 ^b^	4.28 ± 0.40 ^a^	5.28 ± 0.10 ^b^
CLI 194	PC	13.71 ± 0.45 ^a^	60.91 ± 0.09 ^bc^	0.55 ± 0.59	0.77 ± 0.06 ^ab^	1.15 ± 0.06 ^f^	0.73 ± 0.01 ^de^
	SC	4.37 ± 3.07 ^bcde^	0.96 ± 0.41 ^e^	0.52 ± 0.30	0.16 ± 0.01 ^b^	3.55 ±0.21 ^bcd^	5.58 ± 0.38 ^b^
CLI 457	PC	13.74 ± 0.02 ^a^	64.26 ± 0.23 ^a^	0.17 ± 0.01	0.24 ± 0.00 ^b^	1.26 ± 0.01 ^f^	0.62 ± 0.03 ^e^
	SC	2.75 ± 1.12 ^cde^	1.77 ± 0.12 ^e^	0.17 ± 0.03	0.16 ± 0.00 ^b^	4.17 ± 0.02 ^a^	5.67 ± 0.17 ^b^
CLI 512	PC	12.03 ± 0.24 ^ab^	59.11 ± 1.94 ^c^	0.12 ± 0.01	0.24 ± 0.00 ^b^	1.12 ± 0.04 ^f^	0.61 ± 0.01 ^e^
	SC	9.34 ± 5.43 ^abcd^	1.34 ± 0.44 ^e^	0.18 ± 0.02	0.18 ± 0.01 ^b^	3.88 ± 0.27 ^ab^	5.11 ± 0.25 ^b^
CLI 691	PC	13.01 ± 0.14 ^a^	63.16 ± 0.89 ^ab^	0.63 ± 0.01	0.15 ± 0.02 ^b^	1.34 ± 0.01 ^f^	0.63 ± 0.01 ^e^
	SC	10.83 ± 2.16 ^abc^	1.63 ± 0.07 ^e^	0.12 ± 0.03	0.16 ± 0.00 ^b^	3.79 ± 0.09 ^abc^	5.23 ± 0.12 ^b^
CLI 902	PC	13.35 ± 0.12 ^a^	63.94 ± 0.46 ^a^	0.14 ± 0.01	0.26 ± 0.00 ^b^	1.21 ± 0.14 ^f^	1.24 ± 0.03 ^d^
	SC	1.28 ± 0.01 ^e^	1.31 ± 0.04 ^e^	0.16 ± 0.01	0.14 ± 0.01 ^b^	3.54 ± 0.06 ^bcd^	5.18 ± 0.01 ^b^
CLI 1028	PC	15.97 ± 0.47 ^a^	59.06 ± 0.25 ^c^	0.14 ± 0.02	0.24 ± 0.01 ^b^	1.08 ±0.09 ^f^	1.25 ± 0.00 ^d^
	SC	2.09 ± 0.06 ^de^	1.74 ± 0.09 ^e^	0.13 ± 0.00	0.15 ± 0.00 ^b^	2.57 ±0.15 ^e^	5.18 ± 0.23 ^b^
7A-3A	PC	11.58 ± 0.31 ^abc^	59.53 ± 0.22 ^c^	0.12 ± 0.00	0.65 ± 0.00 ^ab^	1.28 ± 0.03 ^f^	0.99 ± 0.01 ^de^
	SC	11.59 ± 0.04 ^abc^	45.31 ± 2.43 ^d^	0.13 ± 0.02	0.22 ± 0.04 ^b^	2.81 ± 0.36 ^de^	2.15 ± 0.20 ^c^
S-04	PC	1.37 ± 0.04 ^e^	1.55 ± 0.01 ^e^	0.11 ± 0.01	0.20 ± 0.04 ^b^	2.89 ± 0.00 ^e^	5.93 ± 0.58 ^a^

Data calculated as mean (*n* = 3) ± standard deviations. Values in the same column with different superscript letters are significantly different (Tukey tests: *p* < 0.05). PC, pure culture; SC, sequential culture. Initial fermentable sugars, maltotriose: 13.75 ± 1.77 g L^−1^; maltose: 59.65 ± 3.59 g L^−1^; glucose: 10.10 ± 4.99 g L^−1^; fructose: 1.23 ± 0.09 g L^−1^.

**Table 4:** Main parameters analysed with CDR of sequentially fermented beers after maturation.

Yeast Strains	Colour EBC	Bitterness IBU	Lactic Acid ppm	SO_2_ ppm	VDKs ppm
CLI 3	12.5 ±0.71 ^abc^	30.00 ± 0.57 ^a^	369.50 ± 70.00 ^a^	1.15 ± 0.21	0.29 ± 0.04 ^abc^
CLI 194	15.00 ± 0.00 ^a^	24.95 ± 0.64 ^ab^	360.00 ± 9.90 ^ab^	≤1.00	0.39 ± 0.00 ^a^
CLI 457	12.00 ± 0.00 ^abc^	34.75 ± 2.05 ^a^	395.50 ± 14.85 ^a^	1.15 ± 0.21	0.16 ± 0.02 ^abc^
CLI 512	13.50 ± 0.71 ^ab^	22.85 ± 10.11 ^ab^	356.50 ± 30.40 ^ab^	≤1.00	0.34 ± 0.00 ^ab^
CLI 691	11.50 ± 0.71 ^bc^	34.35 ± 1.48 ^a^	234.00 ± 7.07 ^b^	1.25 ± 0.07	0.12 ± 0.00 ^bc^
CLI 902	10.33 ± 0.58 ^c^	26.30 ± 2.36 ^ab^	322.00 ± 37.40 ^ab^	≤1.00	0.10 ± 0.03 ^c^
CLI 1028	12.00 ± 0.00 ^abc^	35.20 ± 0.57 ^a^	368.50 ± 17.68 ^a^	1.45 ± 0.64	0.27 ± 0.07 ^abc^
7A-3A	11.50 ± 0.71 ^bc^	26.10 ± 0.28 ^ab^	264.00 ± 12.73 ^ab^	≤1.00	≤0.05 ^c^
S-04	10.00 ± 1.15 ^c^	16.35 ± 1.35 ^c^	304.25 ± 29.03 ^ab^	1.08 ± 0.15	0.32 ± 0.10 ^ab^

Data calculated as mean (*n* = 3) ± standard deviations. Values in the same column with different superscript letters are significantly different (Tukey tests: *p* < 0.05).

**Table 5:** Correlation coefficient, slope, and efficacy of the standard curves in YPD for the different strains.

Yeast Strains	Slope	R^2^	Efficiency (%)
*H. vineae* (CLI 3)	−3.58 ± 0.07	0.997 ± 0.00	90.23 ± 0.02
*H. valbyensis* (CLI 194)	−2.96 ± 0.14	0.993 ± 0.00	118.15 ± 0.08
*M. pulcherrima* (CLI 457)	−3.35 ± 0.04	0.992 ± 0.00	98.90 ± 0.02
*H. gilliermondii* (CLI 512)	−3.33 ± 0.03	0.994 ± 0.00	99.73 ± 0.01
*Z. bailii* (CLI 691)	−4.04 ± 0.15	0.992 ± 0.00	77.02 ±0.04
*T. delbrueckii* (CLI 902)	−3.83 ± 0.05	0.998 ± 0.00	82.56 ± 0.01
*W. anomalus* (CLI 1028)	−3.62 ± 0.11	0.998 ± 0.00	89.27 ± 0.04
*T. delbrueckii* (7A-3A)	−3.67 ± 0.05	0.998 ± 0.00	87.43 ± 0.02
*S. cerevisiae* (S-04)	−3.56 ± 0.08	0.999 ± 0.00	91.14 ± 0.03

Efficiency was calculated using the formula E = (10^−1/slope^)−1.

**Table 6:** Main volatile compounds found in beers fermented sequentially and in S-04 control strain.

Yeast Strains	CLI 3	CLI 194	CLI 457	CLI 512	CLI 691	CLI 902	CLI 1028	7A-3A	S-04
	*H. vineae*	*H. valbyensis*	*M. pulcherrima*	*H. guilliermondii*	*Z. bailii*	*T. delbrueckii*	*W. anomalus*	*T. delbrueckii*	*S. cerevisiae*
**Higher alcohols**									
Isobutanol	19.21 ± 4.84 ^cde^	41.46 ± 1.51 ^b^	60.77 ± 10.62 ^a^	23.23 ± 2.01 ^cde^	27.86 ± 4.13 ^cd^	43.98 ± 2.18 ^b^	30.46 ± 0.04 ^bc^	9.82 ± 1.21 ^e^	28.54 ± 2.29 ^c^
Isoamyl alcohol	59.17 ± 2.37 ^bc^	**110.08 ± 4.64 ^a^**	**107.78 ± 11.95 ^a^**	64.97 ± 9.84 ^bc^	**87.79 ± 7.62 ^ab^**	**113.04 ± 11.44 ^a^**	**73.85 ± 5.79 ^bc^**	55.06 ± 4.72 ^c^	**77.62 ± 5.92 ^bc^**
Methionol	2.18 ± 0.25 ^cd^	5.31 ± 0.62 ^a^	1.33 ± 0.24 ^cd^	2.51 ± 0.83 ^c^	1.14 ± 0.00 ^d^	5.41 ± 0.23 ^a^	1.16 ± 0.10 ^d^	3.94 ± 0.13 ^b^	1.07 ± 0.26 ^d^
β-phenylethanol	10.90 ± 2.64 ^f^	32.76 ± 2.45 ^cde^	21.16 ± 1.90 ^def^	15.88 ± 2.24 ^ef^	58.61 ± 1.85 ^ab^	67.23 ± 2.08 ^a^	40.86 ± 0.79 ^bc^	26.20 ± 2.99 ^cdef^	34.53 ± 8.51 ^cd^
**Esters**									
Ethyl isovalerate	0.12 ± 0.04 ^b^	0.19 ± 0.01 ^b^	0.18 ± 0.02 ^b^	0.13 ± 0.02 ^b^	0.09 ± 0.00 ^b^	0.17 ± 0.03 ^b^	0.15 ± 0.01 ^b^	1.11 ± 0.20 ^a^	0.14 ± 0.02 ^b^
Ethyl butirate	0.16 ± 0.00 ^abc^	0.18 ± 0.00 ^ab^	0.20 ± 0.02 ^a^	0.15 ± 0.01 ^abc^	0.19 ± 0.01 ^a^	0.15 ± 0.01 ^abc^	0.11 ± 0.03 ^c^	nd	0.14 ± 0.01 ^bc^
Isoamyl acetate	0.54 ± 0.02 ^bc^	0.11 ± 0.01 ^c^	**1.19 ± 0.23 ^ab^**	0.25 ± 0.09 ^c^	**1.83 ± 0.03 ^a^**	0.80 ± 0.25 ^bc^	0.04 ± 0.05 ^c^	0.41 ± 0.47 ^bc^	**1.60 ± 0.28 ^a^**
Ethyl hexanoate	0.01 ± 0.02 ^b^	0.05 ± 0.00 ^ab^	0.03 ± 0.01 ^ab^	0.08 ± 0.08 ^ab^	0.03 ± 0.01 ^ab^	0.16 ± 0.02 ^ab^	0.18 ± 0.00 ^a^	0.06 ± 0.01 ^ab^	0.17 ± 0.01 ^a^
Ethyl octanoate	0.03 ± 0.02	0.14 ± 0.02	0.03 ± 0.01	0.14 ± 0.01	0.05 ± 0.00	0.11 ± 0.00	0.19 ± 0.01	0.08 ± 0.00	0.29 ± 0.38
Diethyl succinate	0.52 ± 0.35 ^b^	**4.60 ± 0.72 ^a^**	0.32 ± 0.04 ^b^	0.050 ± 0.41 ^b^	1.05 ± 0.69 ^b^	nd	nd	nd	nd
2-phenylethyl acetate	0.04 ± 0.00 ^b^	0.05 ± 0.01 ^ab^	0.04 ± 0.00 ^b^	0.02 ± 0.00 ^b^	0.10 ± 0.02 ^a^	0.03 ± 0.01 ^b^	0.01 ± 0.00 ^b^	0.04 ± 0.02 ^b^	0.02 ± 0.02 ^b^
**Fatty acids**									
Butyric acid	**2.12 ± 0.19**	1.69 ± 0.01	**2.03 ± 0.05**	1.01 ± 1.43	**2.80 ± 0.00**	1.80 ± 0.00	1.75 ± 0.08	**2.26 ± 0.17**	0.85 ± 0.98
Isobutyric acid	nd	nd	nd	nd	3.05 ± 0.34 ^a^	nd	1.19 ± 0.04 ^bc^	1.31 ± 0.03 ^bc^	1.38 ± 0.20 ^b^
Isovaleric acid	nd	0.19 ± 0.02	nd	0.00 ± 0.00	nd	nd	**6.90 ± 0.34**	**2.85 ± 0.13**	**4.16 ± 4.83**
Hexanoic acid	2.21 ± 0.14 ^abc^	2.97 ± 0.32 ^a^	2.34 ± 0.10 ^ab^	1.98 ± 0.92 ^abc^	1.68 ± 0.10 ^bc^	1.10 ± 0.15 ^cd^	1.69 ± 0.16 ^bc^	0.35 ± 0.10 ^c^	1.25 ± 0.20 ^cd^
Octanoic acid	6.93 ± 0.49 ^ab^	9.58 ± 0.77 ^a^	6.99 ± 0.09 ^ab^	6.60 ± 2.55 ^ab^	4.79 ± 0.53 ^bc^	3.05 ± 0.15 ^cd^	5.22 ± 0.73 ^bc^	0.49 ± 0.39 ^d^	4.83 ± 0.40 ^bc^
Decanoic acid	1.61 ± 0.81 ^ab^	1.88 ± 0.39 ^a^	0.21 ± 0.26 ^bc^	1.03 ± 0.47 ^abc^	0.68 ± 0.15 ^abc^	0.18 ± 0.02 ^bc^	0.82 ± 0.91 ^abc^	0.22 ± 0.11 ^bc^	0.17 ± 0.04 ^c^
**Ketones**									
Diacetyl	0.13 ± 0.03 ^bc^	nd	0.11 ± 0.00 ^c^	0.14 ± 0.02 ^bc^	0.12 ± 0.03 ^c^	**0.39 ± 0.03 ^a^**	nd	**0.37 ± 0.06 ^ab^**	0.10 ± 0.12 ^c^
Acetoin	2.66 ± 1.19 ^ab^	6.72 ± 0.54 ^a^	1.85 ± 0.58 ^b^	4.54 ± 3.26 ^ab^	2.08 ± 0.13 ^b^	1.19 ± 0.42 ^b^	1.44 ± 0.30 ^b^	1.13 ± 0.42 ^b^	1.19 ± 0.42 ^b^
**Phenols**									
Guaiacol	**0.09 ± 0.01 ^bc^**	**0.11 ± 0.00 ^bc^**	**0.09 ± 0.01 ^bc^**	**0.10 ± 0.01 ^bc^**	**0.13 ± 0.00 ^ab^**	**0.12 ± 0.00 ^abc^**	**0.23 ± 0.00 ^a^**	nd	**0.12 ± 0.02 ^bc^**

Data, expressed as mg L^−1^, are calculated as mean (*n* = 3) ± standard deviations; nd = not detected. Compounds above their threshold levels are marked in bold. Values in the same row with different superscript letters are significantly different (Tukey tests: *p* < 0.05).

**Table 7:** Antioxidant capacity of beers fermented sequentially and of the S-04 control strain.

Yeast Strains	Q1	Q2	QT
CLI 3	4.34 ± 0.35 ^abc^	8.93 ± 0.50 ^a^	13.27 ± 0.85 ^ab^
CLI 194	3.47 ± 0.11 ^bc^	7.45 ± 0.14 ^ab^	10.92 ± 0.04 ^abc^
CLI 457	4.44 ± 0.01 ^abc^	8.63 ± 0.53 ^ab^	13.07 ± 0.54 ^ab^
CLI 512	3.56 ± 0.43 ^bc^	8.00 ± 0.21 ^ab^	11.56 ± 0.64 ^abc^
CLI 691	4.58 ± 0.35 ^ab^	7.57 ± 0.85 ^ab^	12.15 ± 0.50 ^abc^
CLI 902	4.34 ± 0.02 ^abc^	8.99 ± 0.76 ^a^	13.33 ± 0.77 ^ab^
CLI 1028	4.80 ± 0.17 ^ab^	8.70 ± 0.12 ^ab^	13.49 ± 0.30 ^a^
7A-3A	3.23 ± 0.13 ^bc^	6.40 ± 0.55 ^b^	9.63 ± 0.42 ^c^
S-04	3.54 ± 0.35 ^bc^	7.64 ± 0.59 ^ab^	11.18 ± 0.94 ^abc^

Data calculated as mean (*n* = 3) ± standard deviations and expressed as millimoles of Trolox equivalents per litre (mmol TE L^−1^). Q1, fast-acting antioxidants; Q2, slow-acting antioxidants; QT, total antioxidants. Values in the same column with different superscript letters are significantly different (Tukey tests: *p* < 0.05).
